# The Potential Consideration of Implicit/Unconscious Gender Bias in Clinical Management Among Patients With Familial Hypercholesterolemia

**DOI:** 10.1002/hsr2.70997

**Published:** 2025-07-07

**Authors:** Kazuhiko Kotani

**Affiliations:** ^1^ Division of Community and Family Medicine Jichi Medical University Tochigi Japan

## Author Contributions

**Kazuhiko Kotani:** conceptualization, investigation, writing – original draft, writing – review and editing; supervision.

## Conflicts of Interest

The author declares no conflicts of interest.

## Transparency Statement

The transparency declaration states that the paper is accurate, honest, and transparent.

To the Editor,

We have read with great interest the study by Kim et al. that showed sex‐based differences in clinical profiles among patients with familial hypercholesterolemia (FH) in Vietnam [1]. This study found that women with FH are diagnosed later and receive a lower treatment intensity than men with FH, who are more likely to smoke and have atherosclerotic cardiovascular diseases [1]. FH is a comparatively popular genetic disorder associated with the likelihood of coronary artery disease, and this trait was also demonstrated in Kim's study [1]. Thus, a nationwide strategy for the prevention of cardiovascular diseases is crucial, and well‐dedicated multifaceted studies are necessary. Even though the findings of that study [1] are similar to the worldwide general trend [2], the results from the registry‐type study are valuable in a specified population of Vietnam, where socio‐cultural characteristics (e.g., norms, roles, thoughts, conceptions, preferences, lifestyles) may differ from those of other countries.

The underlying reasons for the results obtained in Kim's study have not been fully elucidated [1]. The fact that women often have periods of pregnancy and childcare, as well as non‐ownership of vehicles, is one of the reasons contributing to less access to diagnoses and treatment in women with FH [1, 2]. Multiple factors are possibly involved in the findings of the gender difference.

Notably, there might be involvement of implicit/unconscious gender bias (IGB) in clinical management of FH; we would thus further like to raise IGB as a potential reason considered for the gender difference, although there have been few such studies, as IGB is a recent perspective to focus [3, 4, 5]. For instance, an earlier study reported that cardiology physicians view men as being more risk tolerant to cardiovascular tests and may therefore apply angiography more frequently to men than to women [3]. This suggests that IGB by physicians can influence the clinical scenarios in cardiovascular disease‐prone disorders, such as FH, by gender. Women tend to experience atypical chest pain in a suspected case of coronary artery disease, as observed in FH, compared to men [5], and the sharing of such experiences by patients and physicians may also lead to IGB in clinical decision‐making [4].

Importantly, several efforts to ensure equity in clinical management are required. Recognition of IGB by training and recommendation on the use of protocols (standardized regardless of gender) for physicians have been proposed [4, 5]. Collectively, the potential of IGB by physicians and patients may be considered to reveal gender‐based differences in clinical profiles of patients with FH. If IGB is linked with these differences, efforts to realize the equity of screening, clinics, and care should be included in the nationwide strategy for the prevention of cardiovascular diseases in FH. Further research on gender differences in FH, including IGB, is expected.

## Data Availability

Data sharing is not applicable to this article as no data sets were generated or analyzed during the current study.
